# Piezo1 mechanobiology in high myopia and glaucoma: compartment-specific evidence, gaps, and a hypothesis-generating susceptibility framework

**DOI:** 10.3389/fbioe.2026.1925939

**Published:** 2026-10-27

**Authors:** Cong Zhao, Gong Chen, Chun Wang, Huihui Chen, Jingming Shi

**Affiliations:** 1 Department of Ophthalmology, The Second Xiangya Hospital of Central South University, Changsha, China; 2 Hunan Clinical Research Center of Ophthalmic Disease, Changsha, Hunan, China; 3 Clinical Immunology Research Center of Central South University, Changsha, China

**Keywords:** glaucoma, mechanotransduction, myopia, ocular biomechanics, Piezo1, retinal ganglion cell, scleral remodeling, trabecular meshwork

## Abstract

High myopia and glaucoma are clinically distinct but mechanobiologically connected conditions. High myopia is commonly associated with marked axial elongation, posterior-segment stretch, scleral remodeling, and altered posterior-pole and peripapillary biomechanics, whereas glaucoma involves intraocular pressure (IOP)-related outflow stress, optic nerve head (ONH) strain, and retinal ganglion cell (RGC) vulnerability. Both disorders therefore converge on chronic mechanical stress and compartment-specific susceptibility. Piezo1, a mechanically activated cation channel, is well positioned to connect membrane tension, extracellular matrix (ECM) state, Ca^2+^ signaling, reactive oxygen species (ROS)-associated stress responses, and cellular remodeling. This review develops a hypothesis-driven but evidence-bound framework centered on a possible Piezo1-linked mechanobiological susceptibility axis between high myopia and glaucoma. The strongest ocular evidence remains in the trabecular meshwork and conventional outflow pathway, where activation, perturbation, Ca^2+^ signaling, matrix and prostaglandin readouts, and outflow-related assays converge. Evidence in optic nerve head astrocytes is moderate, retinal ganglion cell evidence is emerging, and myopia-related evidence currently supports retinal or posterior-segment stress signaling more strongly than scleral fibroblast-specific ECM causality. Myopia-specific scleral fibroblast evidence and peripapillary-scleral evidence remain exploratory. Critically, current evidence does not demonstrate a continuous Piezo1-dependent causal chain linking scleral remodeling or peripapillary load redistribution to optic nerve head deformation and glaucomatous RGC degeneration. Accordingly, the proposed high-myopia–glaucoma susceptibility axis should be regarded as a hypothesis-generating framework rather than an established causal pathway. This evidence-graded approach distinguishes directly supported tissue-level mechanisms from cross-compartment relationships that require targeted experimental validation.

## 1 Introduction

High myopia and glaucoma are major causes of visual impairment and represent two clinically important challenges in ophthalmology. The global rise in myopia has increased concern about high myopia, excessive axial elongation, and long-term vision-threatening posterior-segment changes, including degenerative changes characteristic of pathologic myopia (Holden et al., 2016; Flitcroft et al., 2019; Ohno-Matsui et al., 2021). Glaucoma remains a leading cause of irreversible blindness worldwide, with IOP, ONH susceptibility, and RGC vulnerability central to disease progression (Tham et al., 2014; Jonas et al., 2017).

Although clinically and anatomically distinct, high myopia and glaucoma converge on a shared mechanobiological problem: ocular tissues must maintain function under chronic mechanical stress and compartment-specific susceptibility (Ethier et al., 2004; Morgan et al., 2012). Following the International Myopia Institute terminology, high myopia refers to a high degree of myopic refractive error, whereas pathologic myopia refers to structural changes of the posterior segment associated with excessive axial elongation, including degenerative complications such as myopic maculopathy and posterior staphyloma (Flitcroft et al., 2019; Ohno-Matsui et al., 2021). In the mechanobiological context considered here, axial elongation, posterior-segment stretch, scleral remodeling, and peripapillary load redistribution are discussed primarily as biomechanical features associated with high myopia, while the term pathologic myopia is reserved for established degenerative structural changes. Glaucoma, in contrast, emphasizes IOP-related outflow stress, ONH strain, and neural vulnerability. Throughout this review, “glaucoma” is used as a broad mechanobiological context when discussing ONH and RGC vulnerability, whereas evidence involving the trabecular meshwork and conventional outflow pathway is interpreted primarily in relation to ocular hypertension and open-angle glaucoma, particularly primary open-angle glaucoma (POAG). The central mechanobiological gap is therefore not whether the eye is mechanically loaded, but which molecular sensors translate tissue-level force into compartment-specific cellular responses.

Recent ocular and non-ocular mechanobiology studies have made Piezo1 a credible candidate mechanism for connecting mechanical loading with cell signaling, although its evidence base varies sharply by tissue. Mechanotransduction links tissue-level forces to cell behavior through coordinated interactions among the lipid membrane, cytoskeleton, focal adhesions, ECM, mechanosensitive ion channels, transcriptional regulators, and inflammatory mediators. Within this network, Piezo1 can convert membrane tension into Ca^2+^ influx and thereby influence cytoskeletal organization, contractility, secretion, gene expression, cell survival, and matrix remodeling in a context-dependent manner (Coste et al., 2010; Kefauver et al., 2020).

The literature is best read as a graded evidence map rather than a single pathway. Direct ocular functional studies, expression surveys, disease models, preprints, and non-ocular analogies each answer different questions. A narrative format is appropriate here because the goal is to place those studies in their tissue context and to separate demonstrated ocular functions from mechanisms that remain plausible but untested.

We propose that high-myopia-related posterior segment remodeling may create a Piezo1-linked mechanobiological susceptibility niche in which chronic tissue stretch, altered scleral and peripapillary load transfer, and glaucoma-relevant IOP stress converge to increase ONH and RGC vulnerability. This model should be interpreted as a compartment-specific susceptibility hypothesis rather than as an established disease pathway centered on Piezo1.

### 1.1 Review scope and evidence appraisal

Evidence appraisal was based on five complementary dimensions: ocular and compartmental relevance, specificity of the evidence for Piezo1, characterization of the mechanical stimulus, demonstration of a tissue-relevant functional outcome, and disease-context or confirmatory validation. Piezo1 specificity was evaluated separately from overall functional relevance. Genetic or molecular perturbation of Piezo1 was given greater evidentiary weight for target attribution than pharmacological manipulation alone. Yoda1-based findings were classified as pharmacological Piezo1 evidence, whereas findings based solely on GsMTx4 were not considered Piezo1-specific because GsMTx4 can modulate multiple mechanosensitive channels. Studies jointly examining Piezo1 and Piezo2 were not attributed specifically to Piezo1 unless the contribution of Piezo1 was independently resolved.

On this basis, evidence within each ocular compartment was organized into five practical levels. Relatively strong evidence required convergent direct ocular evidence combining mechanical stimulation, reasonably Piezo1-specific perturbation, and tissue-relevant functional outcomes. Moderate evidence indicated direct ocular functional evidence with reasonable Piezo1 attribution but incomplete chronic disease, *in vivo*, human, or independent validation. Emerging evidence comprised ocular disease- or injury-related functional findings for which Piezo1 causality, specificity, or replication remained incomplete. Preliminary evidence referred to isolated or insufficiently replicated direct ocular findings requiring confirmation. Exploratory evidence comprised expression-level observations, anatomical or biomechanical plausibility, non-ocular mechanistic analogies, or proposed cross-compartment relationships not directly tested in the relevant ocular tissue. These categories are qualitative organizational tools developed for this narrative review and do not represent a validated grading or risk-of-bias system. The assigned evidence levels reflect the convergence and directness of evidence types, Piezo1 specificity, characterization of the mechanical stimulus, and support from tissue-relevant functional outcomes; they should not be interpreted as formal ratings of individual study quality. Accordingly, “relatively strong” evidence denotes greater convergence of mechanistically relevant evidence within an ocular compartment rather than a high methodological-quality rating of the underlying studies.

### 1.2 What this review adds

Previous reviews have examined Piezo1 in glaucoma, the expression and potential functions of Piezo1 and Piezo2 across ocular tissues and diseases, broader mechanosensitive ion-channel families in glaucoma, and Piezo1–TRPV4 signaling in trabecular meshwork mechanotransduction (Chen et al., 2022; Garcia-Sanchez et al., 2024; Jing et al., 2024; Zhu et al., 2025). Building on these studies, the present review addresses a different question: how strongly and specifically does the available evidence support Piezo1-related mechanotransduction within individual ocular compartments, and to what extent can these compartment-specific findings inform a possible mechanobiological susceptibility interface between high myopia and glaucoma? Rather than treating ocular Piezo1 findings as a uniform evidence base, we systematically distinguish expression-level observations, pharmacological perturbation, genetic or molecular Piezo1-specific perturbation, Piezo1/2-combined findings, preprint-level observations, and non-ocular mechanistic analogies. We also separate experimentally supported tissue-level responses from proposed cross-compartment links. This distinction is particularly important for the high-myopia–glaucoma question because the current evidence is markedly asymmetric: functional evidence is most developed in the trabecular meshwork and conventional outflow pathway, whereas myopia-related Piezo1 evidence is currently centered mainly on retinal or posterior-segment stress responses, and direct evidence linking Piezo1-dependent scleral fibroblast remodeling to peripapillary load transfer, optic nerve head deformation, or glaucomatous retinal ganglion cell degeneration remains lacking. Accordingly, this review does not present Piezo1 as an established causal bridge between high myopia and glaucoma; instead, it provides an evidence-bounded, compartment-resolved framework for distinguishing supported mechanisms from biomechanically plausible hypotheses requiring direct validation.

### 1.3 Literature search and evidence selection

A targeted narrative literature search was performed during manuscript preparation and was subsequently updated through 9 August 2026, using PubMed and Web of Science Core Collection, supplemented by targeted searches in Google Scholar and backward and forward citation tracking. During revision, an additional targeted literature update was conducted through 20 September 2026, to identify newly published studies directly relevant to the review scope and the compartment-specific evidence framework. The structured Piezo-related search covered publications from 1 January 2010 through 9 August 2026. The lower date boundary was selected because Piezo1 and Piezo2 were identified as components of mammalian mechanically activated cation channels in 2010. Search terms incorporated alternative Piezo nomenclature, including “Piezo1,” “Piezo 1,” “Piezo-1,” “FAM38A,” “Piezo2,” “Piezo 2,” “Piezo-2,” and “FAM38B,” in combination with terms describing relevant ocular tissues, diseases, and anatomical compartments, including glaucoma, ocular hypertension, myopia, high myopia, pathologic myopia, trabecular meshwork, Schlemm’s canal, optic nerve head, retinal ganglion cells, retina, sclera, cornea, lens, and ciliary body. Earlier foundational studies of ocular biomechanics, scleral remodeling, aqueous outflow mechanics, and optic nerve head biomechanics published before 2010 were additionally identified through targeted searches and citation tracking when required for mechanistic context.

Peer-reviewed original ocular studies were prioritized for direct evidence appraisal when they reported Piezo1/Piezo2 expression or localization, mechanically induced channel activity, genetic or pharmacological perturbation, tissue-relevant functional responses, aqueous outflow or intraocular pressure regulation, optic nerve head or retinal responses, myopia-related remodeling, or glaucoma-related mechanobiology. Review articles were used to contextualize the field and identify additional primary studies but did not independently increase an evidence level. Non-peer-reviewed reports were considered separately from peer-reviewed evidence and were not used to increase an evidence level. Non-ocular Piezo studies were included selectively as mechanistic analogies and were not treated as direct ocular disease evidence. Meeting abstracts, corrections or errata, clearly off-topic reports, and studies without direct relevance to Piezo-mediated ocular mechanobiology were not used as direct evidence.

Records retrieved from PubMed and Web of Science were checked for duplication using DOI, PMID, and normalized title information. Multiple publications arising from the same underlying experiment, cohort, or dataset were not considered independent replication when evaluating evidence strength. Because this review was designed as a compartment-resolved narrative synthesis rather than a systematic review or quantitative evidence synthesis, no meta-analysis or formal validated risk-of-bias instrument was applied.

Potentially relevant records identified during the primary literature search were independently screened by two authors according to the review scope and evidence-selection criteria. Screening decisions were subsequently cross-checked, and any disagreements were resolved through discussion. Newly identified studies from the revision-stage targeted literature update were assessed using the same evidence-selection criteria. This review was designed as a targeted narrative review rather than a PRISMA-style systematic review.

## 2 Ocular mechanical niches and Piezo1 signaling

### 2.1 Tissue-specific mechanical niches

Here, a mechanical niche refers to the tissue-specific mechanical microenvironment in which ocular cells experience distinct combinations of matrix stiffness, viscoelasticity, fluid shear, stretch, pressure, and cell-ECM coupling. This concept is useful in ophthalmology because ocular tissues are built around different physical demands: the cornea must remain transparent while bearing load, the lens must deform without losing optical clarity, the trabecular meshwork (TM) and Schlemm’s canal must regulate aqueous outflow under pressure and shear, the sclera must preserve globe shape while accommodating growth, and the ONH must support axons within a chronically loaded connective-tissue environment. These compartment-specific mechanical settings help explain why the same Ca^2+^-permeable mechanosensitive channel, such as Piezo1, may produce adaptive signaling in one tissue but stress-related remodeling or injury in another.

Ocular mechanotransduction should therefore be interpreted within compartment-specific mechanical niches rather than as a uniform eye-wide process. This distinction is particularly important for high myopia and glaucoma. In the outflow pathway, TM and Schlemm’s canal cells are exposed to pressure gradients, substrate stiffness, and aqueous shear stress. In high myopia, posterior scleral and peripapillary tissues experience chronic stretch, altered ECM remodeling, and load redistribution associated with axial elongation. In glaucoma, the ONH and peripapillary sclera experience IOP-related stress and strain that influence how mechanical load is transmitted to the lamina cribrosa and adjacent neural tissues (Burgoyne et al., 2005; Sigal et al., 2005; Sigal and Ethier, 2009). These tissue-specific loading environments provide the biomechanical basis for asking whether Piezo1-related signaling links high-myopia-associated posterior segment remodeling with glaucoma-relevant mechanical vulnerability.

This niche-based view is central to interpreting Piezo1 in high myopia and glaucoma. The same Ca^2+^-permeable mechanosensitive channel may support adaptive mechanosensing under transient physiological loading, but become associated with stress-related remodeling or injury when mechanical load is sustained, excessive, or embedded in a diseased matrix environment. Piezo1 effects should therefore be interpreted according to tissue architecture, loading history, disease stage, and downstream signaling context rather than by channel expression alone.

### 2.2 Piezo1 within the ocular mechanosensitive-channel network

The trabecular meshwork (TM) provides the clearest ocular example of Piezo1 functioning within a distributed mechanosensitive network rather than as an isolated sensor. TM cells interact continuously with a specialized ECM and respond to pressure, shear, substrate stiffness, cytoskeletal tension, and changes in contractility and outflow resistance. Piezo1 provides a rapid membrane-tension-sensitive Ca^2+^ entry pathway, whereas TRPV4 and TREK-1 contribute additional Ca^2+^- and pressure-sensitive components of TM mechanotransduction (Ryskamp et al., 2016; Yarishkin et al., 2018; Yarishkin et al., 2021). Direct functional studies further support a role for Piezo1 in conventional outflow physiology (Yarishkin et al., 2021).

These channel-level responses are coupled to focal adhesions, actomyosin organization, transcriptional mechanoregulation, and ECM remodeling. Substrate stiffness modulates YAP/TAZ expression, localization, and ECM-associated gene programs in human TM cells, while increased matrix stiffness can promote YAP/TAZ nuclear localization through focal-adhesion and cytoskeletal rearrangement (Raghunathan et al., 2013; Li et al., 2022). Piezo1 itself can interact with focal-adhesion dynamics and Ca^2+^ entry in a force- and cell-state-dependent manner (Yao et al., 2022). Thus, pressure-, shear-, and stiffness-related inputs are best interpreted as converging on an interconnected Piezo1/TRPV4/TREK-1–cytoskeleton–YAP/TAZ–ECM network rather than on a fixed single-channel hierarchy.

This network perspective is also useful for compartments in which direct Piezo1 evidence remains weaker. In the sclera, for example, the relevant question is not simply whether PIEZO1 is expressed, but whether Piezo1 perturbation alters focal-adhesion signaling, YAP/TAZ activity, matrix turnover, collagen architecture, and tissue stiffness under growth-relevant mechanical loading. Such tissue-resolved experiments are necessary before a scleral Piezo1 mechanism can be placed causally within the myopia-associated remodeling network.

### 2.3 Structural basis and regulatory context

Structural studies established Piezo1 as a large homotrimeric mechanically activated cation channel with a three-bladed architecture that curves the surrounding membrane into a dome-like configuration. Lateral membrane tension can flatten this membrane dome and favor channel opening, providing a physical basis for force-from-lipids gating (Ge et al., 2015; Saotome et al., 2018; Zhao et al., 2018). Channel sensitivity is further shaped by membrane tension, cytoskeletal coupling, and focal-adhesion state rather than by protein structure alone (Lewis and Grandl, 2015; Cox et al., 2016; Yao et al., 2022).

For ocular mechanobiology, the principal implication is that the same Piezo1 architecture is embedded in markedly different mechanical environments. A TM cell exposed to pressure and matrix stiffness, a Schlemm’s canal endothelial cell exposed to shear, an ONH astrocyte undergoing stretch, and a scleral fibroblast embedded in a remodeled ECM may therefore generate different Piezo1-associated Ca^2+^ and downstream responses. ECM composition and inflammatory signaling can further modify Piezo1 mechanosensitivity in non-ocular systems (Lee et al., 2021; Lai et al., 2022). These observations provide mechanistic context for ocular studies but do not define disease function by themselves; tissue architecture, loading history, ECM state, and inflammatory context must be resolved experimentally in each ocular compartment.

## 3 Evidence map for Piezo1 in ocular disease

Current evidence for Piezo1 in ocular disease is uneven across ocular compartments. Expression studies have detected *PIEZO1* and *PIEZO2* across multiple ocular tissues, including the lens, outflow pathway, retina, and other posterior-segment compartments (Doki et al., 2023; Zhu et al., 2023; Zhu et al., 2025). Although this distribution supports broad mechanosensory relevance, expression alone does not establish functional or disease-specific causality. Stronger evidence requires expression to be combined with disease-relevant mechanical stimulation, Ca^2+^ imaging or electrophysiology, Piezo1-specific genetic or molecular perturbation, or pharmacological perturbation interpreted according to tool specificity, and tissue-relevant functional outcomes.

On this basis, evidence in the trabecular meshwork and conventional outflow pathway is currently the most developed. Evidence in optic nerve head astrocytes is moderate, whereas retinal ganglion cell and myopia-related retinal or posterior-segment evidence remains emerging. Direct evidence for myopia-specific Piezo1-dependent scleral fibroblast remodeling and for Piezo1-mediated peripapillary scleral load transfer remains exploratory. Table 1 summarizes these compartment-specific evidence boundaries.

**TABLE 1:** Compartment-specific evidence, gaps, and future experimental priorities for Piezo1 in ocular mechanobiology.

Ocular compartment	Disease/mechanical context	Evidence strength	Current evidence and major gap	Recommended future experiment
Trabecular meshwork	Ocular hypertension/open-angle glaucoma	Relatively strong	Expression; stretch-, pressure-, or Yoda1-induced Ca^2+^ responses; perturbation-sensitive outflow regulation; PGE2/PGF2α release; fibronectin reduction; ferroptosis-related markers under sustained stretch. Long-term *in vivo* therapeutic validation is still incomplete	Chronic outflow models combining cell-specific Piezo1 perturbation with quantitative mechanical and functional validation
Schlemm’s canal	Ocular hypertension/open-angle glaucoma	Preliminary	Preprint-level evidence for candidate Piezo1-related ANGPT2–integrin signaling in outflow regulation; peer-reviewed, cell-type-specific confirmation remains necessary	Peer-reviewed human Schlemm’s canal studies linking Piezo1 perturbation to endothelial permeability and outflow resistance
Optic nerve head astrocytes	Glaucoma	Moderate	Stretch- or Yoda1-induced Ca^2+^ signaling, cytoskeletal remodeling, fibronectin expression, and cell-cycle effects; human disease-stage validation remains incomplete	Chronic strain or ocular-hypertension models with astrocyte-specific Piezo1 perturbation and axonal-support endpoints
Retinal ganglion cells	Glaucoma/mechanical injury	Emerging	Piezo-channel-associated RGC injury evidence; preprint-level Piezo1/2 expression and excitability findings; peer-reviewed hiPSC-derived RGC evidence links PIEZO1 mechanosensitivity to neurite-growth and compartment-specific Ca^2+^ responses; direct *in vivo* causal linkage to chronic glaucomatous RGC death remains unestablished	Cell-specific Piezo1 perturbation linked to RGC survival, axonal integrity, and chronic glaucoma-relevant loading
Retina/posterior segment	Myopia	Emerging	Experimental myopia-related Piezo1 activation and ROS/ferroptosis-associated signaling; model-specific and not equivalent to scleral ECM remodeling	Tissue-resolved studies separating retinal Piezo1 signaling from scleral remodeling and axial-growth effects
Scleral fibroblasts	Myopia	Exploratory	Hypothesized Piezo1 involvement in ECM regulation under axial-elongation-related stretch; direct myopia-specific Piezo1 evidence in scleral fibroblasts remains lacking	Posterior scleral stretch models combining Piezo1 loss-of-function with ECM and biomechanical testing
Peripapillary sclera	Glaucoma	Exploratory	Recent glaucoma-relevant scleral fibroblast evidence supports Piezo1-associated mechanotransduction, but direct evidence for peripapillary localization and Piezo1-dependent load transfer to the ONH remains lacking	Local Piezo1 perturbation linked directly to peripapillary biomechanics and ONH deformation

Evidence strength reflects the current literature and the degree to which ocular expression, mechanical activation, Piezo1 perturbation, and tissue-relevant functional outcomes have been demonstrated. “Exploratory” denotes hypothesis-level or biomechanically plausible evidence without direct compartment-specific Piezo1 causality.

## 4 Piezo1 in myopia-related mechanobiology

Myopia is treated here as a posterior-segment growth and remodeling problem, not only as a refractive state. Visual environment, retinal signaling, choroidal responses, scleral remodeling, and tissue biomechanics interact over time. High myopia provides an axial and biomechanical context in which excessive ocular elongation may be accompanied by posterior-segment and scleral remodeling, whereas pathologic myopia represents a distinct structural disease category characterized by degenerative posterior-segment changes, including myopic maculopathy and posterior staphyloma (Flitcroft et al., 2019; Ohno-Matsui et al., 2021). Both contexts are relevant to posterior-pole biomechanics but should not be used interchangeably. Piezo1 should therefore be positioned within this tissue cascade rather than presented as a free-standing molecular explanation.

### 4.1 Current evidence and tissue-specific interpretation

Classic and contemporary myopia studies describe the sclera as a dynamic load-bearing tissue that undergoes ECM remodeling during ocular growth and experimental myopia. Reported changes include alterations in collagen organization, proteoglycan content, matrix turnover or degradation, and the biomechanical properties of the scleral shell (Gentle et al., 2003; Rada et al., 2006; Harper and Summers, 2015; Troilo et al., 2019). A convincing scleral Piezo1 mechanism would need to connect channel activity with fibroblast Ca^2+^ signaling, collagen synthesis or degradation, crosslinking, tissue stiffness, and whole-eye axial growth.

The available Piezo1-related myopia evidence is currently localized primarily to retinal or posterior-segment stress signaling rather than to direct scleral fibroblast mechanotransduction. Zhong et al. reported increased retinal Piezo1-related signaling in a 4-week form-deprivation myopia model in guinea pigs together with ROS-associated changes (Zhong et al., 2023). This study therefore supports an association between experimental myopia and retinal Piezo1-related oxidative-stress signaling. However, form deprivation represents a disease-model-associated mechanical context rather than an externally imposed and quantitatively controlled retinal-stretch paradigm, and retinal strain was not directly measured. The study consequently does not establish that Piezo1 activation is the initiating mechanical event in myopia, nor does it determine the temporal or causal relationship between retinal Piezo1/ROS signaling and subsequent scleral ECM remodeling (Zhong et al., 2023).

Liu et al. subsequently provided disease-relevant evidence linking PIEZO1-associated signaling with myopia-related outcomes, including refractive change and axial elongation, and further connected PIEZO1-associated activity with retinal ferroptosis-related and oxidative-stress readouts (Liu B. et al., 2025). Pharmacological inhibition with GsMTx4 attenuated several of these phenotypes, and Yoda1-based activation provided complementary pharmacological evidence. Nevertheless, these interventions do not by themselves establish scleral Piezo1-specific causality. GsMTx4 is not fully selective for Piezo1, the principal molecular injury readouts were obtained from retinal tissue, and the study did not directly demonstrate a sequence from scleral mechanical loading to fibroblast Piezo1 activation, altered scleral material properties, and axial elongation. Thus, the study strengthens the biological relevance of Piezo1-associated signaling in experimental myopia without establishing a scleral Piezo1 mechanotransduction pathway.

Taken together, the current retinal findings do not resolve whether Piezo1-associated ROS or ferroptosis signaling is upstream of, parallel to, or compensatory in relation to scleral ECM remodeling. Each interpretation remains biologically plausible. An upstream model would require evidence that retinal Piezo1 perturbation alters downstream scleral remodeling and axial growth; a parallel model would predict retinal and scleral mechanotransductive responses to a shared myopiagenic environment without a direct causal link between them; and a compensatory model would require temporal evidence that retinal Piezo1 activation emerges in response to, rather than before, established ocular remodeling. Distinguishing among these possibilities will require longitudinal experiments with tissue-resolved Piezo1 perturbation and concurrent measurements of retinal signaling, scleral biomechanics, ECM remodeling, refraction, and axial length.

To place this uncertainty in a broader mechanistic context, any future scleral Piezo1 mechanism should be evaluated within an established non-Piezo1 mechanotransduction network rather than as an isolated candidate sensor. Collagen-binding integrins provide a direct interface between the scleral extracellular matrix and fibroblasts: α1β1-and α2β1-integrin expression is dynamically regulated during experimental myopia, and these receptors localize to scleral fibroblast focal adhesions (McBrien et al., 2006). More recent work has directly linked scleral ECM stiffness to an integrin α1β1–F-actin–YAP–COL1A1 mechanotransduction axis, providing a mechanism by which altered matrix mechanics can be translated into collagen remodeling in myopic sclera (Liu X. et al., 2025).

Direct scleral mechanotransduction has also been demonstrated for the related mechanosensitive channel Piezo2, which was reported to mediate stretch-induced scleral fibroblast differentiation through Ca^2+^-dependent RelA/RhoA signaling in experimental myopia (Yuan et al., 2026).

ECM turnover constitutes another established component of myopic scleral remodeling. In experimental myopia, selected matrix metalloproteinases and tissue inhibitors of metalloproteinases are dynamically regulated during minus-lens compensation and recovery, with these changes temporally associated with axial elongation and altered scleral mechanical properties (Siegwart and Norton, 2005). Complementary remodeling pathways include scleral hypoxia and HIF-1α-associated signaling (Wu et al., 2018), as well as THBS1/TSP-1-associated scleral remodeling during myopia development (Chen et al., 2024). Importantly, these pathways should not be interpreted as established downstream targets of Piezo1; rather, they define experimentally supported components of the scleral remodeling network against which a future Piezo1 mechanism should be tested.

Together, these studies clarify why direct myopia-specific scleral Piezo1 evidence remains limited and is classified here as exploratory despite strong biomechanical plausibility. Myopic sclera already contains experimentally supported integrin–focal-adhesion, cytoskeletal/YAP, matrix-turnover, hypoxia-associated, and matricellular remodeling pathways, but current studies have not established where Piezo1 lies within this network. In particular, no myopia study has yet demonstrated, within the same experimental sequence, a defined scleral mechanical stimulus, Piezo1-specific activation or genetic perturbation in scleral fibroblasts, downstream mechanotransductive or ECM changes, altered scleral material properties, and consequent axial-growth outcomes. Moreover, an isolated scleral fibroblast response in culture may not reproduce the geometry, viscoelastic loading, collagen architecture, or growth-related cues of the posterior pole. Establishing a convincing scleral Piezo1 mechanism will therefore require tissue-specific loss-of-function or rescue experiments that connect these levels rather than inference from channel expression or retinal stress readouts alone.

### 4.2 High myopia as a Piezo1-linked glaucoma susceptibility context

High myopia may be more than a parallel disease context; it may create a posterior-segment mechanical environment that modifies glaucoma susceptibility. Axial elongation, scleral remodeling, posterior pole deformation, and peripapillary load redistribution could alter how IOP-related stress is transmitted to the ONH and adjacent neural tissues. Within this setting, Piezo1-related signaling may provide one molecular route through which chronic stretch, oxidative stress, ferroptosis-associated responses, and matrix remodeling influence tissue vulnerability.

Against this evidence background, the high-myopia–glaucoma connection should be treated as a mechanobiological susceptibility axis requiring tissue-resolved validation rather than as an established scleral Piezo1 pathway.

The useful next step is model resolution. Experimental myopia studies should distinguish readouts from the retina, choroid, posterior segment as a whole, and scleral fibroblasts. *In vivo* studies should include axial length, refractive error, posterior segment imaging, scleral stiffness, and collagen architecture; cell and tissue models need posterior scleral stretch, fibroblast-specific *PIEZO1* knockdown or conditional deletion, live-cell Ca^2+^ imaging, ECM transcript and protein measurements, and mechanical testing of scleral strips. Such designs would allow Piezo1 to be classified as a causal regulator, a stress-responsive marker, or one component of a broader mechanosensitive network.

This framing protects the myopia section from overreach. The presence of a mechanosensitive channel in a mechanically loaded tissue is not sufficient to establish disease causality. For myopia, the evidence chain must extend to growth-relevant endpoints: refractive shift, axial elongation, posterior pole structure, scleral material behavior, and ECM architecture. A Piezo1-associated change in ROS or ferroptosis-related markers may be biologically important, but it constitutes evidence for a myopia mechanism only when anchored to ocular growth and scleral remodeling outcomes.

## 5 Piezo1 in glaucoma-related mechanobiology

Glaucoma provides an important ocular setting for investigating Piezo1 because IOP-related loading, conventional outflow resistance, ONH strain, and neural susceptibility are central mechanobiological considerations. However, the disease relevance of the available evidence differs by compartment. In particular, studies of the trabecular meshwork and conventional outflow pathway are most directly relevant to ocular hypertension and open-angle glaucoma, including POAG, whereas ONH astrocyte, RGC, and peripapillary-scleral findings address broader glaucoma-relevant mechanical and neurodegenerative processes. These compartments should therefore not be merged into a single Piezo1 pathway. Consistent with this distinction, a mechanosensitive ion-channel gene survey suggested potential involvement of Piezo-related mechanosensory pathways in POAG, although such association-level evidence does not establish Piezo1-specific disease causality (Liu et al., 2023).

In the conventional outflow pathway, the TM, juxtacanalicular tissue, Schlemm’s canal inner wall, and their associated ECM are central to aqueous outflow resistance generation (Acott and Kelley, 2008; Overby et al., 2009; Tamm, 2009). TM cells are exposed to pressure gradients, aqueous flow, stretch or tissue distortion, cell-ECM mechanical coupling, phagocytic function, and ongoing ECM turnover (Acott and Kelley, 2008; Tamm, 2009; Vranka et al., 2015). In addition, substrate stiffness can modulate YAP/TAZ-related signaling and ECM-associated gene programs in human TM cells (Raghunathan et al., 2013; Li et al., 2022). A membrane-tension-sensitive channel embedded in that environment could support physiological outflow adaptation under transient load and contribute to injury under prolonged stress. Accordingly, the TM-focused evidence reviewed below should be interpreted primarily as evidence for conventional-outflow mechanotransduction with particular relevance to ocular hypertension and open-angle glaucoma, rather than as evidence applicable uniformly across all glaucoma subtypes.

### 5.1 Trabecular meshwork and aqueous outflow

The most direct ocular evidence for Piezo1 concerns the TM and conventional outflow pathway, but the underlying studies should be attributed precisely. Uchida et al. showed that mechanical stretch or Yoda1 activation induced Piezo1-dependent Ca^2+^ influx and extracellular PGE2 release in human trabecular meshwork cells (Uchida et al., 2021). In that study, a single uniaxial stretch with a nominal 30% chamber extension produced an approximately 20% extension of the cell-culture surface, and stretch-associated lipid mediators were quantified at 10, 30, and 60 min after stimulation (Uchida et al., 2021). This provides human TM cell-level evidence for mechanotransduction, arachidonic acid–PGE2 signaling, and contractility-related responses; it should not be interpreted as a direct demonstration of aqueous outflow facility regulation.

The stronger tissue-level outflow evidence comes from Yarishkin et al., who investigated TM mechanotransduction using quantitatively defined pressure-, shear-, and perfusion-based paradigms. Whole-cell pressure pulses of 25 mmHg for 1.5 s and excised-patch pressure pulses of −80 mmHg for 500 ms were used to evoke mechanically activated currents; laminar shear stress of 0.5 dyn/cm^2^ was applied for 10 s during Ca^2+^ imaging, and paired enucleated mouse eyes were initially perfused at 12 mmHg for 60 min before sequential pressure steps spanning 5–17 mmHg (Yarishkin et al., 2021). In human TM cells, pressure stimulation evoked a rapid Piezo1-sensitive current that was inhibited by Ruthenium Red or GsMTx4 and attenuated by *PIEZO1* knockdown. The same study linked Piezo1 activation to Ca^2+^ signaling, focal cell-matrix contact remodeling, and modulation of the trabecular component of aqueous outflow, including measurements in paired enucleated mouse eyes using the iPerfusion system (Yarishkin et al., 2021). Complementary *in vivo* pharmacological evidence was provided by Morozumi et al., who reported that topical Yoda1 reduced IOP in mice and that Piezo1 activation in human trabecular meshwork cells was associated with reduced fibronectin expression and increased MMP-2 expression (Morozumi et al., 2021).

Subsequent work by Morozumi et al. added a matrix-associated biochemical layer by showing that pharmacological Piezo1 activation with Yoda1 in human trabecular meshwork cells was associated with reduced fibronectin and increased PGF2α secretion through an arachidonic-acid-associated cascade (Morozumi et al., 2022). Liu et al. further extended the sustained-stress side of the TM evidence using 20% cyclic strain at 0.125 Hz (8 s/cycle) in human trabecular meshwork cells (Liu et al., 2024). Ca^2+^ influx was assessed after 10 min of stretch, whereas 12 h of stretching was associated with increased ROS accumulation and lipid peroxidation, increased ACSL4, and decreased GPX4 expression; these ferroptosis-associated changes were reversed or attenuated by GsMTx4 or *PIEZO1* knockdown (Liu et al., 2024). Pharmacological Piezo1 activation with Yoda1 reproduced key Ca^2+^ and lipid-peroxidation-related responses, further supporting the involvement of Piezo1 in this sustained-stress phenotype (Liu et al., 2024). Together, these studies broaden the TM evidence base from rapid mechanosensitive Ca^2+^ signaling and outflow-related responses to matrix regulation and sustained-stress-associated injury phenotypes.

The mechanical-loading paradigms used across TM studies differed substantially in stimulus type and experimental time scale, ranging from acute pressure, shear, or single-stretch paradigms to intermediate-duration cyclic loading and sustained low-frequency strain (Uchida et al., 2021; Yarishkin et al., 2021; Zhu et al., 2021; Liu et al., 2024). This heterogeneity should be considered when comparing rapid Piezo1-associated channel activation and Ca^2+^ signaling with longer-duration matrix, oxidative-stress, and injury-associated responses. The principal loading parameters are summarized in Table 2.

**TABLE 2:** Quantitative mechanical-loading paradigms in representative ocular Piezo studies.

Study	Ocular model	Mechanical paradigm	Quantitative loading	Piezo manipulation	Key readout/interpretation
Uchida et al., 2021	Primary human trabecular meshwork (TM) cells	Single uniaxial stretch	Nominal 30% chamber extension; actual culture-surface extension ≈20%; 1 way/s with a 3.0-s pause; lipid mediators measured at 10, 30, and 60 min after stretch	*PIEZO1* siRNA; Yoda1	Ca^2+^ influx; arachidonic acid and PGE_2_ release; contraction-related responses
Zhu et al., 2021	Human and mouse primary TM cells; enucleated mouse eyes	Cyclic stretch; cellular indentation; *ex vivo* pressure perfusion	20% cyclic stretch at 1 Hz for 3 h; sequential 1-μm indentations with 6-μm test indentation; 8 mmHg acclimation for 45 min followed by 4.5–16.5 mmHg pressure steps	*PIEZO1* shRNA; Yoda1; GsMTx4	Piezo1 expression and mechanically activated current; outflow facility; ocular compliance
Yarishkin et al., 2021	Primary human TM cells; paired enucleated mouse eyes	Pressure clamp; laminar shear; *ex vivo* perfusion	Whole-cell pressure pulse: 25 mmHg for 1.5 s; excised patch: −80 mmHg for 500 ms; shear stress: 0.5 dyn/cm^2^ for 10 s for Ca^2+^ imaging; paired-eye perfusion: 12 mmHg for 60 min, then 5–17 mmHg	*PIEZO1* shRNA; Yoda1; GsMTx4	Mechanically activated currents; Ca^2+^ signaling; focal cell–matrix contacts; aqueous outflow facility
Liu et al., 2021	Primary mouse optic nerve head (ONH) astrocytes	PDMS-based 3-D stretch	10% strain produced by 100-μm membrane displacement at 100 μm/s; *Piezo1* mRNA assessed after 2 h and immunocytochemical expression after 24 h	Yoda1 (100–500 nM; 300 nM for 72-h reactivity assays)	Piezo1 expression; Ca^2+^; F-actin redistribution; fibronectin
Liu et al., 2024	Human TM cells	Low-frequency cyclic stretch	20% strain at 0.125 Hz (8 s/cycle); 10 min for Ca^2+^ measurement and 12 h for ROS, lipid peroxidation, ferroptosis-related markers, and viability	*PIEZO1* knockdown; GsMTx4; Yoda1	Ca^2+^ influx; ROS; lipid peroxidation; ACSL4; GPX4; cell viability
Chen et al., 2025	hiPSC-derived retinal ganglion cells	Magnetic nanoparticle/field-mediated mechanical guidance	External permanent magnets, each 2292 ± 58.7 Gauss; magnetic field applied from the day after cell seeding during a 4-day culture paradigm	*PIEZO1* shRNA; Yoda1 10 μM; Dooku1 10 μM	Neurite elongation and alignment; somatic and neuritic Ca^2+^ activity; specialized magnetic-force model rather than a glaucoma-like loading paradigm
Zhong et al., 2023	Guinea-pig form-deprivation myopia (FDM) retina	Disease-model-associated posterior-segment mechanical context	4-week FDM; no externally imposed or directly quantified retinal strain	GsMTx4 10 μM, 5 μL intravitreally every 3 days	Refraction; axial length; retinal Piezo1 expression; ROS; mechanical load inferred from disease context rather than directly measured

Numerical loading parameters are reported only when explicitly stated in the original studies. Disease-model-associated mechanical exposure is distinguished from externally imposed, quantitatively controlled loading. TM, trabecular meshwork; ONH, optic nerve head; FDM, form-deprivation myopia; PGE_2_, prostaglandin E_2_; ROS, reactive oxygen species; ACSL4, acyl-CoA synthetase long-chain family member 4; GPX4, glutathione peroxidase 4; hiPSC, human induced pluripotent stem cell.

Taken together, the available TM studies are consistent with a context-dependent spectrum of Piezo1-associated responses rather than a directly demonstrated adaptive-to-injurious switch. Acute or shorter-duration stimulation has been associated with Ca^2+^ signaling, secretion, focal-adhesion remodeling, and outflow-related responses, whereas sustained mechanical stress in different experimental systems has been associated with oxidative stress, lipid peroxidation, ferroptosis-related changes, and cellular injury. Because these observations derive from different models, loading paradigms, and experimental time scales, it remains unclear whether Piezo1 itself transitions from an adaptive to an injurious mechanism or whether distinct mechanical and matrix contexts produce different downstream outcomes. The adaptive-versus-injurious concept should therefore be regarded as an interpretive framework requiring direct longitudinal validation.

This evidence should still be placed in the wider outflow network. Glaucomatous TM shows increased stiffness (Last et al., 2011) and ECM dysregulation (Vranka et al., 2015; Keller and Peters, 2022), substrate stiffness modulates YAP/TAZ and ECM-associated genes (Raghunathan et al., 2013; Li et al., 2022), and single-cell or tissue-level studies have highlighted outflow-pathway heterogeneity (van Zyl et al., 2020). Human TM stiffness, ECM, and outflow-pathway heterogeneity studies provide useful mechanical context for testing Piezo1 alongside TRPV4, TREK-1, integrins, focal adhesions, and YAP/TAZ rather than as an isolated channel (Raghunathan et al., 2013; Gagen et al., 2014; Vranka et al., 2015; Ryskamp et al., 2016; Yarishkin et al., 2018; van Zyl et al., 2020; Li et al., 2022).

A 2025 bioRxiv preprint proposed that Piezo1 in Schlemm’s canal activates an autocrine Angiopoietin-2-integrin signaling loop that may regulate IOP (Kiyota et al., 2025). Because this evidence is preprint-level, it should remain preliminary until peer-reviewed confirmation is available. It is nevertheless useful because it separates Schlemm’s canal endothelial mechanobiology from TM cell mechanobiology and strengthens the rationale for cell-type-specific outflow studies.

### 5.2 Optic nerve head astrocytes and retinal ganglion cells

ONH astrocytes provide a second glaucoma-relevant Piezo1 compartment. In primary mouse optic nerve head astrocytes, a PDMS-based 3-D mechanical-stretch paradigm used a 100-μm membrane displacement at 100 μm/s to generate approximately 10% strain (Liu et al., 2021). *Piezo1* mRNA was assessed after 2 h of stretch, whereas immunocytochemical Piezo1 expression was assessed after 24 h (Liu et al., 2021). Yoda1 induced dose-dependent Ca^2+^ responses at 100–500 nM and, at 300 nM over 72 h, was associated with F-actin redistribution and increased fibronectin expression (Liu et al., 2021). These quantitative experimental conditions are summarized in Table 2. Wan et al. further linked Piezo1 to ONH astrocyte cell-cycle progression (Wan et al., 2023). Genetic association work on *PIEZO1* mechanoreceptor variants provides additional background, but it should not be treated as causal evidence for glaucomatous tissue injury (Baxter et al., 2020). These findings support a moderate level of evidence for glial mechanosensing, while leaving human disease-stage mapping and chronic ocular hypertension models as necessary validation steps.

RGC evidence remains more tentative. Piezo channel activity has been implicated in retinal ganglion cell damage, but this should not be written as proof that Piezo1 directly causes glaucomatous RGC death (Morozumi et al., 2020). A preprint-level study reported Piezo1/2 expression in RGCs and found that Piezo1 activation increased light-evoked action potential frequency and spontaneous dark activity in mouse retinal explants (Sripinun et al., 2024). These functional readouts are important because altered excitability could interact with mitochondrial stress, ROS amplification, calpain activity, survival, and axon integrity. Altered excitability is not the same as glaucomatous RGC death. More recently, Chen et al. used hiPSC-derived RGCs together with magnetic nanoparticles and an external magnetic field to examine force-guided neurite growth (Chen et al., 2025). PIEZO1 was detected in both somatic and neuritic compartments; pharmacological inhibition with Dooku1 enhanced neurite elongation, whereas Yoda1 increased somatic Ca^2+^ activity without independently promoting neurite outgrowth. This study provides peer-reviewed human-cell evidence linking PIEZO1-dependent mechanosensitivity to neurite-growth responses, although the specialized magnetic-force paradigm should not be interpreted as a direct model of chronic glaucoma-related mechanical loading.

A useful glaucoma model should therefore keep glial and neuronal questions separate before reconnecting them. For ONH astrocytes, the central readouts are glial state, cytoskeletal organization, fibronectin and ECM remodeling, inflammatory tone, and axonal-support capacity. For RGCs, the relevant bridge runs from channel expression and excitability to mitochondrial stress, Ca^2+^-dependent protease activity, survival, and axonal integrity. Only after those cell-type-specific responses are established should the field ask whether Piezo1 coordinates a broader neuroglial response to chronic IOP-related strain.

Disease timing is equally important. A Piezo1-dependent astrocyte response early after stretch could represent an adaptive attempt to reorganize the ONH microenvironment, whereas a persistent response during chronic IOP exposure could contribute to ECM stiffening, inflammatory signaling, or impaired axonal support. RGC excitability changes may also occur before irreversible degeneration. Longitudinal models that measure glial state, light-evoked function, spontaneous activity, axon integrity, and cell survival in the same experimental series would help distinguish early mechanosensing from late neurodegenerative injury.

### 5.3 Peripapillary sclera: a biomechanically plausible but under-tested compartment

The peripapillary sclera is mechanically coupled to the ONH and helps shape how IOP-related stress and strain are transmitted to the lamina cribrosa and adjacent neural tissues (Burgoyne et al., 2005). It is therefore a logical compartment for mechanotransduction studies in glaucoma. Recent evidence has extended Piezo1-related mechanotransduction to scleral fibroblasts in a glaucoma-relevant mechanical context, with cyclic strain, pharmacological modulation, and PIEZO1 knockdown linking Piezo1 activity to myofibroblast differentiation and ECM-related responses (Hu et al., 2026). However, peripapillary-scleral Piezo1 evidence remains exploratory because direct localization and functional testing in the glaucomatous peripapillary sclera, particularly in relation to tissue-scale load transfer and ONH deformation, are still lacking. The next useful evidence would come from human donor tissue, posterior pole organ culture, or animal glaucoma models with local fibroblast-specific Piezo1 perturbation and tissue-scale deformation readouts.

## 6 Lessons from non-ocular mechanobiology: mechanisms to test in ocular systems

Non-ocular studies are most useful here as mechanism-generating analogies rather than as direct ocular evidence. Three examples suggest specific experiments for ocular compartments. First, epithelial stretch can activate Piezo1-dependent proliferative programs (Gudipaty et al., 2017), raising testable questions about whether deformation-dependent Piezo1 signaling contributes to adaptive or maladaptive remodeling in TM cells or scleral fibroblasts. Second, vascular studies show that Piezo1 senses shear-related forces and contributes to endothelial architecture and vascular regulation (Li et al., 2014; Wang et al., 2016); the closest ocular analogue is Schlemm’s canal and the conventional outflow endothelium, where shear-sensitive Piezo1 signaling could be tested against outflow and endothelial-barrier readouts. Third, Piezo1-mediated sensing of cyclical force can regulate innate immune responses (Solis et al., 2019), providing a rationale for examining whether chronic mechanical loading modifies Piezo1-dependent inflammatory signaling in ONH astrocytes or other glaucoma-relevant ocular immune and glial environments.

ECM and fibrosis studies provide a complementary set of hypotheses for the TM and sclera. Matrix stiffness, integrin–actomyosin coupling, and YAP/TAZ signaling are established regulators of mechanically responsive cell states (Engler et al., 2006; Dupont et al., 2011; Humphrey et al., 2014), while Piezo1-associated signaling has been linked to fibroblast activation, inflammatory amplification, and matrix deposition or degradation across several non-ocular models (Fu et al., 2021; Lee et al., 2021; Swain et al., 2022; Zhao et al., 2022; Feng et al., 2024; Zheng et al., 2024; Zhang et al., 2025). Matrix composition can also modify Piezo1 responses to shear stress (Lai et al., 2022). These findings support testing Piezo1–ECM coupling in TM and scleral models, but they do not justify transferring a profibrotic, catabolic, or inflammatory phenotype directly from another organ to the eye. Ocular validation therefore requires compartment-specific mechanical loading, Piezo1-specific perturbation, and tissue-relevant functional and biomechanical readouts.

## 7 Piezo1-linked susceptibility axis and tissue-resolved evidence map

High myopia and glaucoma should not be interpreted as components of a single Piezo1-centered disease cascade. In particular, no continuous experimental evidence currently links Piezo1-dependent scleral remodeling to peripapillary biomechanical alteration, ONH deformation, and subsequent glaucomatous RGC degeneration. Rather, high-myopia-related posterior-segment remodeling may create a mechanically susceptible environment in which Piezo1-related signaling intersects with glaucoma-relevant IOP stress, ONH deformation, conventional outflow regulation, and RGC vulnerability. Figure 1 depicts this hypothesis-driven susceptibility-axis model across a strain-dominant high-myopia context, a central Piezo1-linked susceptibility interface, and a pressure-dominant glaucoma context. Figure 2 provides a complementary tissue-resolved appraisal of the evidence, distinguishing relatively strong findings in the TM and conventional outflow pathway from preliminary evidence in Schlemm’s canal, moderate evidence in ONH astrocytes, emerging evidence involving RGCs and the myopic posterior segment, and exploratory evidence in the posterior and peripapillary sclera.

**FIGURE 1:**
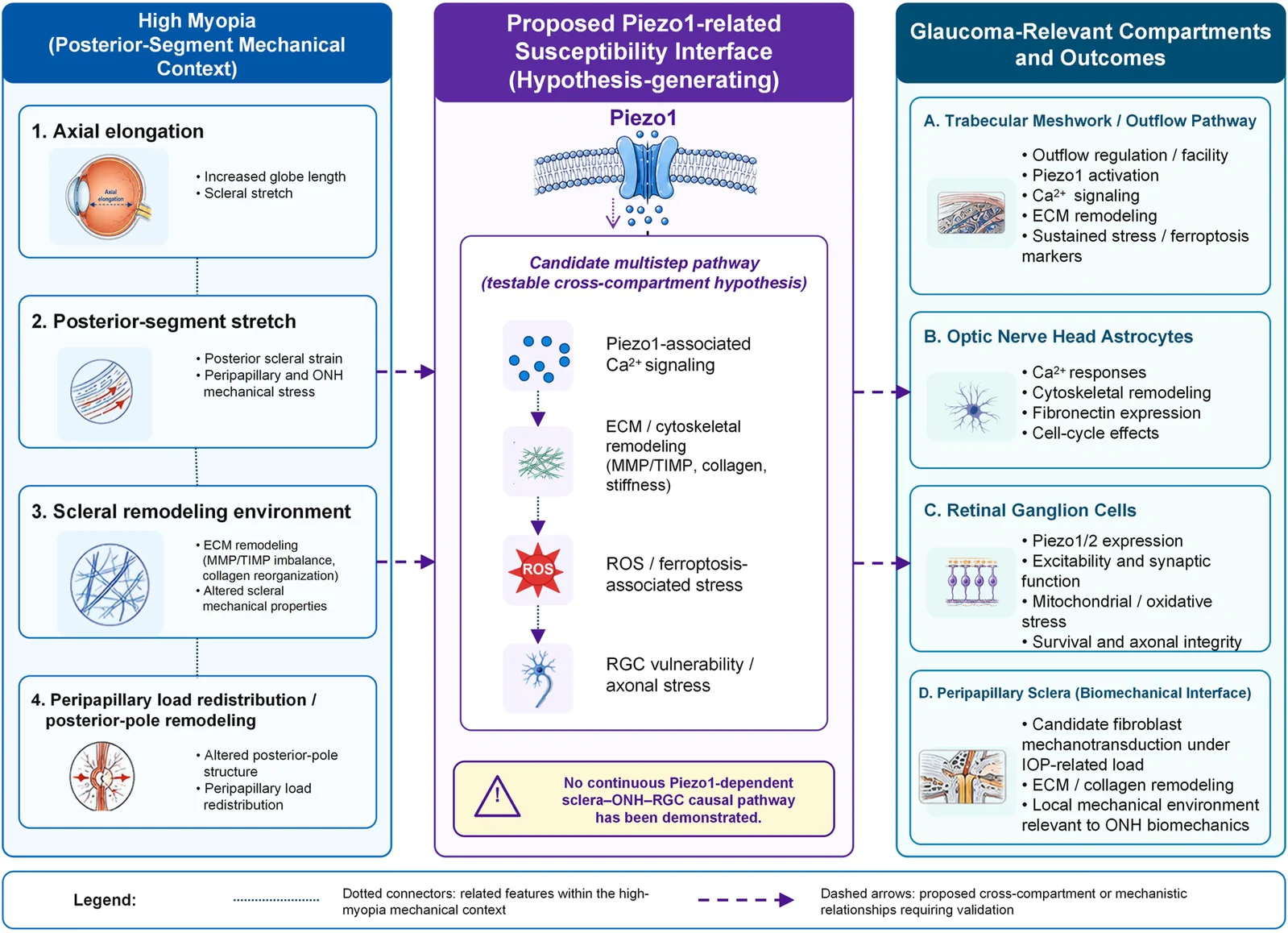
Evidence-bounded conceptual framework for a proposed Piezo1-related mechanobiological susceptibility interface between high myopia and glaucoma. The left panel summarizes related mechanical features of high myopia, including axial elongation, posterior-segment stretch, scleral remodeling, and peripapillary/posterior-pole load redistribution. Dotted connectors indicate related features within the high-myopia mechanical context and do not imply a directional causal sequence. The central panel presents a hypothesis-generating candidate multistep pathway involving Piezo1-associated Ca^2+^ signaling, extracellular-matrix (ECM)/cytoskeletal remodeling, ROS/ferroptosis-associated stress, and retinal ganglion cell (RGC) vulnerability or axonal stress. The right panel summarizes glaucoma-relevant compartments and outcomes involving the trabecular meshwork/conventional outflow pathway, optic nerve head (ONH) astrocytes, RGCs, and the peripapillary sclera. Purple dashed arrows indicate proposed cross-compartment or mechanistic relationships that require direct validation. Importantly, these proposed links should not be interpreted as demonstrating a continuous Piezo1-dependent causal pathway from scleral remodeling through ONH biomechanical alteration to RGC injury.

**FIGURE 2:**
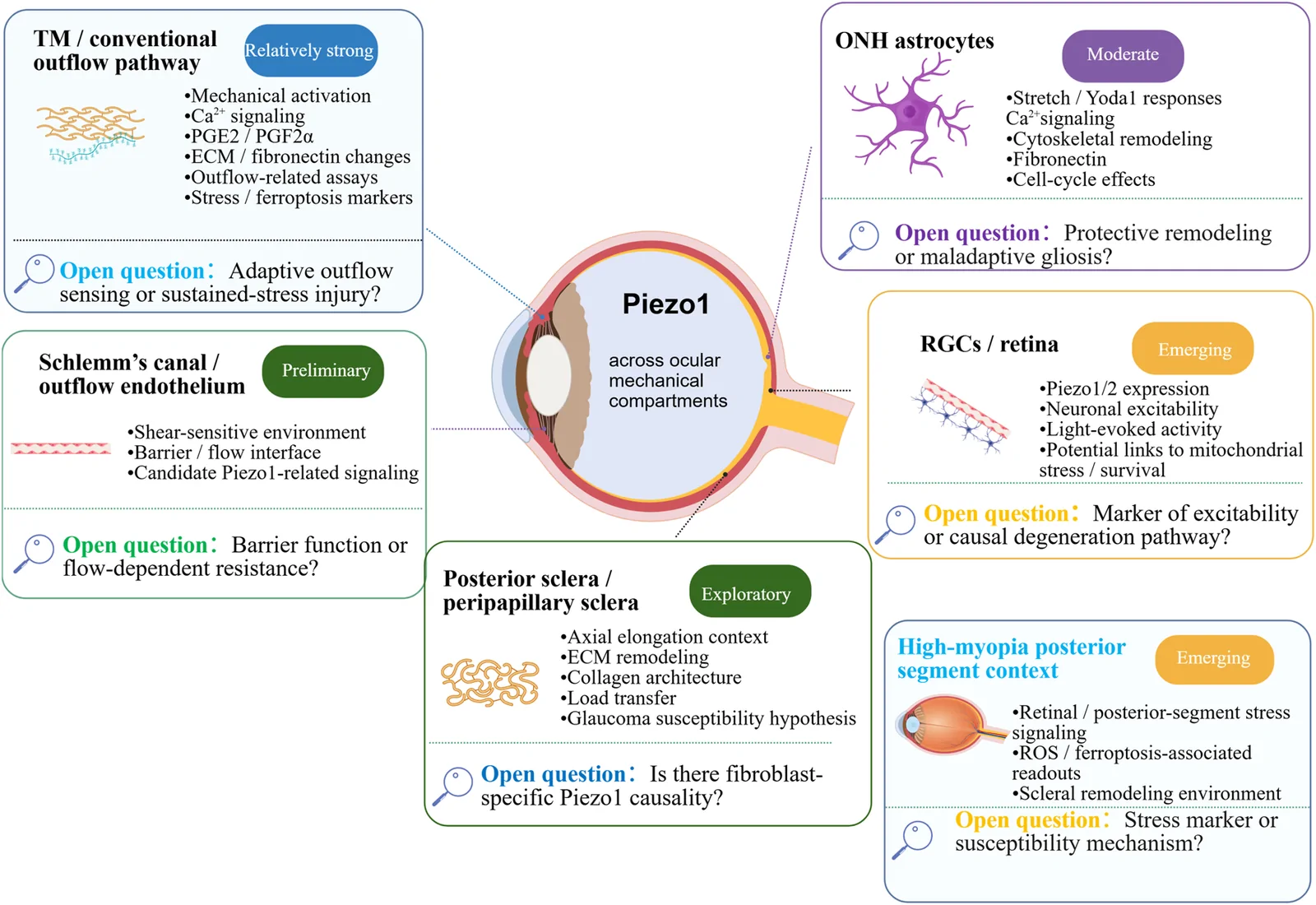
Tissue-resolved evidence map of ocular Piezo1 mechanobiology. This schematic summarizes compartment-specific evidence strength and unresolved questions for Piezo1-related mechanobiology across ocular tissues. Evidence from the TM and conventional outflow pathway is currently the strongest, including mechanical activation, Ca^2+^ signaling, prostaglandin- and ECM-associated readouts, outflow-related assays, and markers associated with oxidative stress and ferroptosis. Evidence in Schlemm’s canal and outflow endothelium remains preliminary and is framed around endothelial barrier function and flow-dependent outflow resistance. ONH astrocyte evidence is moderate, whereas evidence involving RGCs, the retina, and the posterior segment in high myopia remains emerging. Posterior scleral and peripapillary scleral evidence remains exploratory, particularly with respect to myopia-specific or peripapillary-localized Piezo1 causality, collagen architecture, tissue-scale load transfer, and ONH deformation. Overall, this map highlights the compartment-specific nature of current Piezo1 evidence and identifies tissue contexts in which further functional validation is most needed. Created with BioGDP.com.

Together, Figures 1, 2 are intended to distinguish hypothesis generation from compartment-specific evidence appraisal.

## 8 Discussion and translational outlook

Building on the compartment-specific evidence summarized above, the following discussion focuses on unresolved mechanistic interpretations, translational limitations, and experimental priorities for Piezo1-related ocular mechanobiology.

### 8.1 Key controversies and competing interpretations

Several unresolved issues complicate the interpretation of Piezo1-related findings in high myopia and glaucoma. These competing interpretations are not necessarily mutually exclusive, but they indicate that the consequences of Piezo1 activation depend on ocular compartment, loading history, extracellular matrix state, disease stage, and downstream signaling context.

The first controversy concerns whether Piezo1 signaling is adaptive or injurious. In the trabecular meshwork, current studies collectively support both outflow-related adaptive responses and stress-associated injury phenotypes (Uchida et al., 2021; Yarishkin et al., 2021; Morozumi et al., 2022; Liu et al., 2024). However, these findings were obtained using different experimental models, mechanical stimuli, pharmacological conditions, and exposure durations. The proposed adaptive-versus-injurious continuum should therefore be regarded as a cross-study interpretation rather than as a directly demonstrated biphasic switch. It remains unclear whether Piezo1 itself changes from an adaptive to an injurious mechanism or whether a common mechanosensory response produces different outcomes according to matrix stiffness, oxidative state, inflammatory signaling, and stimulus duration.

A second issue is whether Piezo1 is a dominant ocular mechanotransducer or one component of a broader mechanosensitive network. Trabecular meshwork and outflow-pathway mechanobiology also involve TRPV4 and TREK-1 (Ryskamp et al., 2016; Yarishkin et al., 2018), as well as stiffness-responsive YAP/TAZ and extracellular matrix programs (Raghunathan et al., 2013; Li et al., 2022). Current evidence supports the functional relevance of these pathways, but direct sequential, compensatory, or epistatic relationships between them and Piezo1 remain insufficiently defined. Piezo1 may therefore act as a necessary driver in some ocular compartments, a partially redundant sensor in others, or one regulatory node within a distributed mechanotransduction network.

A third controversy concerns whether Piezo1 provides a causal molecular bridge between high myopia and glaucoma or whether the two conditions primarily share a biomechanically vulnerable tissue environment. Axial elongation and posterior scleral remodeling are important biomechanical features associated with high myopia, whereas pathologic myopia additionally encompasses degenerative structural changes of the posterior segment, including myopic maculopathy and posterior staphyloma (Flitcroft et al., 2019; Ohno-Matsui et al., 2021). Peripapillary scleral properties and optic nerve head geometry, in turn, influence the transmission of intraocular-pressure-related stress and strain (Burgoyne et al., 2005; Sigal et al., 2005; Sigal and Ethier, 2009). Piezo1-related findings in experimental myopia support retinal or posterior-segment stress signaling, including reactive-oxygen-species- and ferroptosis-associated responses (Zhong et al., 2023; Liu B. et al., 2025). However, these studies do not establish a continuous causal chain from Piezo1-dependent scleral fibroblast remodeling to peripapillary load redistribution, optic nerve head deformation, and glaucomatous retinal ganglion cell loss. The proposed high-myopia–glaucoma susceptibility axis should therefore remain a testable mechanobiological framework rather than an established Piezo1-centered disease pathway.

A final issue is the distinction between expression, functional responsiveness, and disease causality. Differential expression of *PIEZO1* and *PIEZO2* in ocular tissues establishes biological plausibility but does not by itself demonstrate a disease-relevant function (Zhu et al., 2023). Likewise, Ca^2+^ or electrophysiological responses to mechanical stimulation or Yoda1 demonstrate functional responsiveness but do not necessarily establish a role in chronic disease progression. In retinal ganglion cells, Piezo-channel-associated injury and changes in light-evoked or spontaneous activity following Piezo1 stimulation have been reported, but these findings are not equivalent to a demonstrated causal role in chronic glaucomatous retinal ganglion cell death (Morozumi et al., 2020; Sripinun et al., 2024). Similarly, retinal oxidative-stress or ferroptosis-associated findings in experimental myopia cannot be directly interpreted as evidence of scleral extracellular-matrix causality (Zhong et al., 2023; Liu B. et al., 2025). Resolving these alternatives will require longitudinal, cell-type-specific loss-of-function and rescue studies linking Piezo1 perturbation to tissue biomechanics, axial elongation, optic nerve head deformation, axonal integrity, and retinal ganglion cell survival.

Taken together, these competing interpretations favor a compartment- and context-dependent model. The central question is not simply whether Piezo1 is expressed or activated, but when, where, and under which mechanical and molecular conditions its activity becomes necessary for disease-relevant remodeling or injury.

### 8.2 Therapeutic translation

Pharmacological tools require careful interpretation. Yoda1 and related agonists can probe channel activation but do not reproduce every physiological mechanical stimulus (Syeda et al., 2015). GsMTx4 is useful for mechanosensitive-channel inhibition but is not fully Piezo1-specific (Bae et al., 2011). Tubeimoside I and other inhibitors may offer additional experimental options, yet their ocular pharmacokinetics, toxicity, and cell-type effects remain to be established (Liu et al., 2020). Genetic approaches and rescue experiments will be necessary to separate Piezo1-specific effects from broader mechanosensitive-channel biology.

For glaucoma, local targeting could theoretically aim to preserve adaptive TM mechanosensing while preventing sustained-stress injury. For example, downstream modulation of ferroptosis, oxidative stress, inflammatory sensitization, or ECM stiffness might be safer than complete Piezo1 blockade. For ONH or RGC protection, delivery becomes more difficult because the relevant cells are posterior and neural. Intravitreal or periocular approaches may provide access but require rigorous safety testing.

For myopia, therapeutic translation depends on first establishing whether Piezo1 in posterior segment tissues is causally related to axial elongation or scleral remodeling. If the strongest evidence remains retinal or model-specific, Piezo1 is best viewed as a research target for mechanobiology-guided myopia studies rather than a near-term clinical target.

Therapeutic targeting of Piezo1 is attractive but difficult. Inhibition could be beneficial where excessive Piezo1 signaling promotes abnormal Ca^2+^ entry, ferroptosis, gliosis, inflammation, or maladaptive ECM remodeling. However, global inhibition may disrupt physiological mechanotransduction in the TM, endothelium, erythrocytes, and other tissues. Human disorders caused by gain-of-function *PIEZO1* variants and vascular studies highlight the systemic safety issues that could accompany broad channel modulation (Albuisson et al., 2013; Wang et al., 2016).

The eye offers translational advantages because local delivery can reduce systemic exposure. Topical, periocular, sub-Tenon, and intravitreal routes can be considered depending on the target compartment, although each has anatomical and pharmacokinetic constraints (Kompella et al., 2021). For glaucoma, future strategies may focus on restoring TM mechanoadaptation, preventing sustained-stress injury, or modulating downstream pathways rather than simply blocking Piezo1. For myopia, the priority is not immediate treatment translation but proof of causality in posterior segment and scleral models.

### 8.3 Experimental priorities

The evidence map points to a small set of practical experimental priorities. Ocular studies need to pair mechanical loading with Piezo1-sensitive perturbation and real-time Ca^2+^ imaging. Tissue-specific models are needed to separate retinal, choroidal, scleral, TM, Schlemm’s canal, ONH astrocyte, and RGC effects. Single-cell and spatial transcriptomics should be integrated with mechanical phenotyping to identify disease-stage-specific Piezo1-positive cell states. Pharmacologic studies should account for tool specificity, especially when broad mechanosensitive-channel inhibitors such as GsMTx4 are used.

Future studies should prioritize experimental designs that connect molecular signaling to tissue mechanics. In the TM, this means combining controlled pressure or stretch paradigms with outflow measurements, ECM stiffness, traction force, Ca^2+^ imaging, and Piezo1 perturbation. In ONH astrocytes, it means connecting Ca^2+^ and cytoskeletal responses to ECM remodeling, inflammatory phenotype, and axonal support. In RGCs, it means determining whether Piezo1-related excitability changes translate into survival or degeneration in chronic ocular hypertension models. Future experimental designs should therefore report mechanical loading quantitatively wherever possible, including strain magnitude, pressure or shear level, loading frequency, and exposure duration, together with tissue-specific Piezo1 perturbation and functional endpoints.

In myopia research, the most important gap is scleral specificity. Studies should distinguish retina, choroid, scleral fibroblasts, and whole-eye mechanical outcomes. Posterior scleral stretch systems, tissue-engineered scleral ECM, and *in vivo* conditional models would allow the field to test whether Piezo1 regulates fibroblast phenotype, MMP/TIMP balance, collagen architecture, and axial elongation. Without these experiments, the scleral Piezo1 model remains plausible but incomplete.

Single-cell and spatial transcriptomics can help resolve tissue heterogeneity, but expression atlases require functional validation. Mechanotransduction is a dynamic process, so the strongest future studies will combine spatial expression, cell-type-specific perturbation, mechanical loading, live signaling readouts, and tissue-level biomechanics.

This roadmap also clarifies why precision ocular biomechanics should not rely on a single-marker strategy. Optical coherence tomography (OCT)-based deformation mapping and digital volume correlation can quantify ONH tissue strain *in vivo*, and recent work shows that myopia, glaucoma, eye movement, and IOP-related loading can alter ONH biomechanical susceptibility (Midgett et al., 2019; Chuangsuwanich et al., 2023; Chuangsuwanich et al., 2024). These mechanical phenotypes could be combined with IOP fluctuation, outflow facility, axial length progression, posterior segment imaging, and single-cell or spatial profiling of outflow and retinal tissues (van Zyl et al., 2020; Zhu et al., 2023). In such a framework, Piezo1 would be one layer in a compartment-specific risk model rather than a general disease label. The translational priority is therefore to pair molecular profiling with a measurable mechanical or functional phenotype within the same ocular compartment.

A tissue-resolved roadmap also changes how negative results should be interpreted. If Piezo1 perturbation fails to alter axial length in an *in vivo* myopia model but changes retinal stress signaling, that would argue against a direct scleral growth mechanism while preserving a posterior-segment signaling role. If perturbation changes TM Ca^2+^ responses but not outflow facility, the channel may be present without being functionally decisive for resistance regulation under that condition. Clear compartment-specific endpoints make such outcomes informative rather than merely disappointing.

### 8.4 Mechanical memory and long-term remodeling

Chronic mechanical exposure may leave a durable cellular state after the initial stimulus has changed, a phenomenon often described as mechanical memory. Cells exposed to a particular stiffness or force history may retain altered cytoskeletal organization, transcriptional activity, or differentiation tendency even after mechanical conditions are modified (Yang et al., 2014). For ocular disease, this idea is attractive because both myopia and glaucoma involve long time scales, repeated mechanical loading, and tissue remodeling that may persist after a transient stimulus.

At present, the ocular Piezo1 literature is not strong enough to state that Piezo1 writes an epigenetic memory in myopic sclera or glaucomatous TM. Piezo1 may participate in mechanical-memory-like processes if sustained Ca^2+^ signaling, cytoskeletal remodeling, YAP/TAZ activity, ECM stiffness, and chromatin regulation become linked in a stable feedback loop. Such a loop would be consistent with general mechanobiology, but a direct Piezo1-epigenetic pathway in eye disease has not been established.

In myopia, a mechanical-memory framework could help explain why scleral remodeling may continue across time even as visual environment, growth signals, and mechanical loading vary. A scleral fibroblast that has been exposed to chronic stretch, hypoxia, inflammatory mediators, or altered matrix stiffness may not return immediately to a baseline state. It may retain changes in contractility, matrix production, matrix degradation, or mechanosensitive signaling. Whether Piezo1 contributes to this persistence is a testable question that would require time-course experiments, washout or recovery designs, chromatin assays, and tissue-level mechanical measurements.

In glaucoma, mechanical memory could be relevant to the TM and ONH. TM cells exposed to chronic ECM stiffening or pressure-related stretch may acquire persistent contractile, fibrotic, or stress-sensitive states. ONH astrocytes exposed to repeated strain may similarly retain a reactive phenotype. Yet the therapeutic implication should be stated carefully. It is premature to propose combined Piezo1 inhibition and epigenetic therapy as a near-term ocular treatment. A stronger conclusion is that mechanical memory should be investigated as a future research direction linking chronic load, Piezo1-related Ca^2+^ signaling, cytoskeletal remodeling, and durable tissue phenotype.

### 8.5 Limitations of the evidence base and review methodology

Several limitations of the current evidence base constrain mechanistic interpretation and clinical translation. Much of the available functional evidence derives from cultured cells, *ex vivo* preparations, or animal models that only partially reproduce chronic human ocular biomechanics and disease-stage heterogeneity. Direct human and longitudinal evidence remains limited, particularly for the ONH, RGC, posterior scleral, and peripapillary compartments. In addition, no validated Piezo1-based ocular biomarker has yet been established for monitoring tissue deformation, disease progression, or treatment response. These limitations should be considered when extrapolating experimental findings toward clinical application.

The present review also has methodological limitations. It was designed as a targeted, compartment-resolved narrative review rather than as a systematic review. The primary literature screening was independently performed by two authors, with screening decisions subsequently cross-checked. An additional targeted literature update was conducted during revision using the same evidence-selection criteria. Therefore, no PRISMA-style flow diagram or formal systematic-review workflow was applied.

Language and publication bias also cannot be excluded. The search relied primarily on major international databases and English-language search terminology, which may underrepresent relevant studies that are less readily indexed or reported in other languages. Positive or mechanistically coherent findings may also be more likely to be published than negative or null results, particularly in a relatively small and heterogeneous literature. Finally, no validated study-level quality or risk-of-bias instrument was applied. The evidence categories used throughout this Review therefore reflect convergence and directness of evidence, Piezo1 specificity, characterization of mechanical loading, and tissue-relevant functional support rather than formal methodological-quality scores. A compartment classified as having “relatively strong” evidence should consequently not be interpreted as containing uniformly high-quality studies.

The figures and tables require the same caution. Figure 1 presents an evidence-bounded, hypothesis-generating framework for a possible Piezo1-related mechanobiological susceptibility interface between high myopia and glaucoma, whereas Figure 2 summarizes compartment-specific evidence strength and unresolved questions. Table 1 summarizes the current evidence boundaries across ocular compartments, while Table 2 provides a quantitative comparison of representative mechanical-loading paradigms reported in ocular Piezo studies. Together, these visual and tabular frameworks are intended to distinguish supported observations from proposed cross-compartment or mechanistic relationships without implying equal certainty across tissues or a proven Piezo1-mediated causal pathway from high myopia to glaucoma.

### 8.6 Concluding perspective

TM studies provide the clearest functional chain, with expression, activation, perturbation, biochemical readouts, and outflow-related outcomes. ONH astrocyte studies provide moderate evidence for glial mechanosensing and remodeling, whereas RGC studies provide emerging evidence related to excitability and stress vulnerability.

A rigorous evidence-bound framework should therefore treat Piezo1 as a candidate mechanosensory interface rather than an established molecular bridge between high myopia and glaucoma. Myopia-related Piezo1 evidence remains emerging and is currently centered mainly on retinal or posterior-segment stress responses, whereas direct evidence for myopia-specific Piezo1-dependent scleral fibroblast remodeling remains limited. More importantly, current studies do not establish a continuous causal sequence in which Piezo1-dependent scleral remodeling alters peripapillary load transfer, produces ONH deformation, and subsequently drives glaucomatous RGC degeneration. The high-myopia–glaucoma susceptibility axis proposed here should therefore be regarded as a hypothesis-generating framework. Its validation will require longitudinal, tissue-specific Piezo1 perturbation studies that simultaneously relate scleral and peripapillary biomechanics to ONH strain, axonal integrity, and RGC survival.

## References

[B1] Acott T. S. Kelley M. J. (2008). Extracellular matrix in the trabecular meshwork. Exp. Eye Res. 86 (4), 543–561. 10.1016/j.exer.2008.01.013 18313051 PMC2376254

[B2] Albuisson J. Murthy S. E. Bandell M. Coste B. Louis-Dit-Picard H. Mathur J. (2013). Dehydrated hereditary stomatocytosis linked to gain-of-function mutations in mechanically activated PIEZO1 ion channels. Nat. Commun. 4, 1884. 10.1038/ncomms2899 23695678 PMC3674779

[B3] Bae C. Sachs F. Gottlieb P. A. (2011). The mechanosensitive ion channel Piezo1 is inhibited by the peptide GsMTx4. Biochemistry 50 (29), 6295–6300. 10.1021/bi200770q 21696149 PMC3169095

[B4] Baxter S. L. Keenan W. T. Athanas A. J. Proudfoot J. A. Zangwill L. M. Ayyagari R. (2020). Investigation of associations between Piezo1 mechanoreceptor gain-of-function variants and glaucoma-related phenotypes in humans and mice. Sci. Rep. 10 (1), 19013. 10.1038/s41598-020-76026-0 33149214 PMC7643131

[B5] Burgoyne C. F. Downs J. C. Bellezza A. J. Suh J. K. Hart R. T. (2005). The optic nerve head as a biomechanical structure: a new paradigm for understanding the role of IOP-related stress and strain in the pathophysiology of glaucomatous optic nerve head damage. Prog. Retin Eye Res. 24 (1), 39–73. 10.1016/j.preteyeres.2004.06.001 15555526

[B6] Chen Y. Su Y. Wang F. (2022). The Piezo1 ion channel in glaucoma: a new perspective on mechanical stress. Hum. Cell 35 (5), 1307–1322. 10.1007/s13577-022-00738-w 35767143

[B7] Chen J. Ikeda S. I. Yang Y. Zhang Y. Ma Z. Liang Y. (2024). Scleral remodeling during myopia development in mice eyes: a potential role of thrombospondin-1. Mol. Med. 30 (1), 25. 10.1186/s10020-024-00795-x 38355399 PMC10865574

[B8] Chen I. T. Chu P. H. Hu S. H. Chen S. K. Chiao C. C. Yang T. C. (2025). Magnetically guided neurite outgrowth modulated by PIEZO1 in hiPSC-derived retinal ganglion cells. Mater. Today Bio 35, 102446. 10.1016/j.mtbio.2025.102446 41209706 PMC12590149

[B9] Chuangsuwanich T. Tun T. A. Braeu F. A. Yeoh C. H. Y. Chong R. S. Wang X. (2023). How Myopia and Glaucoma influence the biomechanical susceptibility of the optic nerve head. Invest. Ophthalmol. Vis. Sci. 64 (11), 12. 10.1167/iovs.64.11.12 37552032 PMC10411647

[B10] Chuangsuwanich T. Tun T. A. Braeu F. A. Wang X. Chin Z. Y. Panda S. K. (2024). Adduction induces large optic nerve head deformations in subjects with normal-tension glaucoma. Br. J. Ophthalmol. 108 (4), 522–529. 10.1136/bjo-2022-322461 37011991

[B11] Coste B. Mathur J. Schmidt M. Earley T. J. Ranade S. Petrus M. J. (2010). Piezo1 and Piezo2 are essential components of distinct mechanically activated cation channels. Science 330 (6000), 55–60. 10.1126/science.1193270 20813920 PMC3062430

[B12] Cox C. D. Bae C. Ziegler L. Hartley S. Nikolova-Krstevski V. Rohde P. R. (2016). Removal of the mechanoprotective influence of the cytoskeleton reveals PIEZO1 is gated by bilayer tension. Nat. Commun. 7, 10366. 10.1038/ncomms10366 26785635 PMC4735864

[B13] Doki Y. Nakazawa Y. Sukegawa M. Petrova R. S. Ishida Y. Endo S. (2023). Piezo1 channel causes lens sclerosis via transglutaminase 2 activation. Exp. Eye Res. 237, 109719. 10.1016/j.exer.2023.109719 37951336

[B14] Dupont S. Morsut L. Aragona M. Enzo E. Giulitti S. Cordenonsi M. (2011). Role of YAP/TAZ in mechanotransduction. Nature 474 (7350), 179–183. 10.1038/nature10137 21654799

[B15] Engler A. J. Sen S. Sweeney H. L. Discher D. E. (2006). Matrix elasticity directs stem cell lineage specification. Cell 126 (4), 677–689. 10.1016/j.cell.2006.06.044 16923388

[B16] Ethier C. R. Johnson M. Ruberti J. (2004). Ocular biomechanics and biotransport. Annu. Rev. Biomed. Eng. 6, 249–273. 10.1146/annurev.bioeng.6.040803.140055 15255770

[B17] Feng X. Li S. Wang S. Meng Y. Zheng S. Liu C. (2024). Piezo1 mediates the degradation of cartilage extracellular matrix in malocclusion-induced TMJOA. Oral Dis. 30 (4), 2425–2438. 10.1111/odi.14615 37184045

[B18] Flitcroft D. I. He M. Jonas J. B. Jong M. Naidoo K. Ohno-Matsui K. (2019). IMI - Defining and classifying myopia: a proposed set of standards for clinical and epidemiologic studies. Invest. Ophthalmol. Vis. Sci. 60 (3), M20–m30. 10.1167/iovs.18-25957 30817826 PMC6735818

[B19] Fu Y. Wan P. Zhang J. Li X. Xing J. Zou Y. (2021). Targeting mechanosensitive Piezo1 alleviated renal fibrosis through p38MAPK-YAP pathway. Front. Cell Dev. Biol. 9, 741060. 10.3389/fcell.2021.741060 34805150 PMC8602364

[B20] Gagen D. Faralli J. A. Filla M. S. Peters D. M. (2014). The role of integrins in the trabecular meshwork. J. Ocul. Pharmacol. Ther. 30 (2-3), 110–120. 10.1089/jop.2013.0176 24266581 PMC3991962

[B21] Garcia-Sanchez J. Lin D. Liu W. W. (2024). Mechanosensitive ion channels in glaucoma pathophysiology. Vis. Res. 223, 108473. 10.1016/j.visres.2024.108473 39180975 PMC11398070

[B22] Ge J. Li W. Zhao Q. Li N. Chen M. Zhi P. (2015). Architecture of the mammalian mechanosensitive Piezo1 channel. Nature 527 (7576), 64–69. 10.1038/nature15247 26390154

[B23] Gentle A. Liu Y. Martin J. E. Conti G. L. McBrien N. A. (2003). Collagen gene expression and the altered accumulation of scleral collagen during the development of high myopia. J. Biol. Chem. 278 (19), 16587–16594. 10.1074/jbc.M300970200 12606541

[B24] Gudipaty S. A. Lindblom J. Loftus P. D. Redd M. J. Edes K. Davey C. F. (2017). Mechanical stretch triggers rapid epithelial cell division through Piezo1. Nature 543 (7643), 118–121. 10.1038/nature21407 28199303 PMC5334365

[B25] Harper A. R. Summers J. A. (2015). The dynamic sclera: extracellular matrix remodeling in normal ocular growth and myopia development. Exp. Eye Res. 133, 100–111. 10.1016/j.exer.2014.07.015 25819458 PMC4379420

[B26] Holden B. A. Fricke T. R. Wilson D. A. Jong M. Naidoo K. S. Sankaridurg P. (2016). Global prevalence of Myopia and High Myopia and Temporal Trends from 2000 through 2050. Ophthalmology 123 (5), 1036–1042. 10.1016/j.ophtha.2016.01.006 26875007

[B27] Hu D. Jiang J. Zheng X. Wang C. Qiu C. Wang X. (2026). Piezo1 promotes scleral ECM remodeling via mechanical strain-induced fibroblast differentiation. J. Vis. Exp. 234, 71340. 10.3791/71340 42611751

[B28] Humphrey J. D. Dufresne E. R. Schwartz M. A. (2014). Mechanotransduction and extracellular matrix homeostasis. Nat. Rev. Mol. Cell Biol. 15 (12), 802–812. 10.1038/nrm3896 25355505 PMC4513363

[B29] Jiang S. Li H. Zhang L. Mu W. Zhang Y. Chen T. (2025). Generic Diagramming Platform (GDP): a comprehensive database of high-quality biomedical graphics. Nucleic Acids Res. 53 (D1), D1670–d1676. 10.1093/nar/gkae973 39470721 PMC11701665

[B30] Jing L. Liu K. Wang F. Su Y. (2024). Role of mechanically-sensitive cation channels Piezo1 and TRPV4 in trabecular meshwork cell mechanotransduction. Hum. Cell 37 (2), 394–407. 10.1007/s13577-024-01035-4 38316716

[B31] Jonas J. B. Aung T. Bourne R. R. Bron A. M. Ritch R. Panda-Jonas S. (2017). Glaucoma. Lancet 390 (10108), 2183–2193. 10.1016/s0140-6736(17)31469-1 28577860

[B32] Kefauver J. M. Ward A. B. Patapoutian A. (2020). Discoveries in structure and physiology of mechanically activated ion channels. Nature 587 (7835), 567–576. 10.1038/s41586-020-2933-1 33239794 PMC8477435

[B33] Keller K. E. Peters D. M. (2022). Pathogenesis of glaucoma: extracellular matrix dysfunction in the trabecular meshwork-A review. Clin. Exp. Ophthalmol. 50 (2), 163–182. 10.1111/ceo.14027 35037377 PMC9199435

[B34] Kiyota N. Deb D. K. Thomson B. R. Quaggin S. E. (2025). Piezo1 activates an autocrine Angiopoietin-2-Integrin signaling loop in schlemm's canal to regulate intraocular pressure. bioRxiv. 10.1101/2025.10.24.683742 41279337 PMC12633313

[B35] Kompella U. B. Hartman R. R. Patil M. A. (2021). Extraocular, periocular, and intraocular routes for sustained drug delivery for glaucoma. Prog. Retin Eye Res. 82, 100901. 10.1016/j.preteyeres.2020.100901 32891866 PMC8317199

[B36] Lai A. Thurgood P. Cox C. D. Chheang C. Peter K. Jaworowski A. (2022). Piezo1 response to shear stress is controlled by the components of the extracellular matrix. ACS Appl. Mater. Interfaces 14 (36), 40559–40568. 10.1021/acsami.2c09169 36047858

[B37] Last J. A. Pan T. Ding Y. Reilly C. M. Keller K. Acott T. S. (2011). Elastic modulus determination of normal and glaucomatous human trabecular meshwork. Invest. Ophthalmol. Vis. Sci. 52 (5), 2147–2152. 10.1167/iovs.10-6342 21220561 PMC3080174

[B38] Lee W. Nims R. J. Savadipour A. Zhang Q. Leddy H. A. Liu F. (2021). Inflammatory signaling sensitizes Piezo1 mechanotransduction in articular chondrocytes as a pathogenic feed-forward mechanism in osteoarthritis. Proc. Natl. Acad. Sci. U. S. A. 118 (13), e2001611118. 10.1073/pnas.2001611118 33758095 PMC8020656

[B39] Lewis A. H. Grandl J. (2015). Mechanical sensitivity of Piezo1 ion channels can be tuned by cellular membrane tension. Elife 4, e12088. 10.7554/eLife.12088 26646186 PMC4718726

[B40] Li J. Hou B. Tumova S. Muraki K. Bruns A. Ludlow M. J. (2014). Piezo1 integration of vascular architecture with physiological force. Nature 515 (7526), 279–282. 10.1038/nature13701 25119035 PMC4230887

[B41] Li H. Raghunathan V. Stamer W. D. Ganapathy P. S. Herberg S. (2022). Extracellular Matrix stiffness and TGFβ2 regulate YAP/TAZ activity in human trabecular meshwork cells. Front. Cell Dev. Biol. 10, 844342. 10.3389/fcell.2022.844342 35300422 PMC8923257

[B42] Liu S. Pan X. Cheng W. Deng B. He Y. Zhang L. (2020). Tubeimoside I antagonizes Yoda1-Evoked Piezo1 channel activation. Front. Pharmacol. 11, 768. 10.3389/fphar.2020.00768 32523536 PMC7261832

[B43] Liu J. Yang Y. Liu Y. (2021). Piezo1 plays a role in optic nerve head astrocyte reactivity. Exp. Eye Res. 204, 108445. 10.1016/j.exer.2021.108445 33465396 PMC7946740

[B44] Liu W. W. Kinzy T. G. Cooke Bailey J. N. Xu Z. Hysi P. Wiggs J. L. (2023). Mechanosensitive ion channel gene survey suggests potential roles in primary open angle glaucoma. Sci. Rep. 13 (1), 15871. 10.1038/s41598-023-43072-3 37741866 PMC10517927

[B45] Liu K. Xu J. Yang R. Wang F. Su Y. (2024). Ion channel Piezo1 induces ferroptosis of trabecular meshwork cells: a novel observation in the pathogenesis in primary open angle glaucoma. Am. J. Physiol. Cell Physiol. 327 (6), C1591–c1603. 10.1152/ajpcell.00173.2024 39466179 PMC11684867

[B46] Liu B. Yao X. Huang Q. Shi Z. Wei J. Li S. (2025a). Sodium fluoride promotes myopia progression via the activation of the ferroptosis pathway by PIEZO1 and pharmacological targeting PIEZO1 represents an innovative approach for myopia treatment. Cell Biol. Toxicol. 41 (1), 64. 10.1007/s10565-025-10020-y 40175653 PMC11965261

[B47] Liu X. Yuan Y. Wu Y. Zhu C. Liu Y. Ke B. (2025b). Extracellular Matrix stiffness modulates Myopia scleral remodeling through Integrin/F-Actin/YAP axis. Invest. Ophthalmol. Vis. Sci. 66 (2), 22. 10.1167/iovs.66.2.22 39918477 PMC11809450

[B48] McBrien N. A. Metlapally R. Jobling A. I. Gentle A. (2006). Expression of collagen-binding integrin receptors in the mammalian sclera and their regulation during the development of myopia. Invest. Ophthalmol. Vis. Sci. 47 (11), 4674–4682. 10.1167/iovs.05-1150 17065473

[B49] Midgett D. E. Quigley H. A. Nguyen T. D. (2019). *In vivo* characterization of the deformation of the human optic nerve head using optical coherence tomography and digital volume correlation. Acta Biomater. 96, 385–399. 10.1016/j.actbio.2019.06.050 31279161 PMC6717668

[B50] Morgan I. G. Ohno-Matsui K. Saw S. M. (2012). Myopia. Lancet 379 (9827), 1739–1748. 10.1016/s0140-6736(12)60272-4 22559900

[B51] Morozumi W. Inagaki S. Iwata Y. Nakamura S. Hara H. Shimazawa M. (2020). Piezo channel plays a part in retinal ganglion cell damage. Exp. Eye Res. 191, 107900. 10.1016/j.exer.2019.107900 31874142

[B52] Morozumi W. Aoshima K. Inagaki S. Iwata Y. Nakamura S. Hara H. (2021). Piezo 1 is involved in intraocular pressure regulation. J. Pharmacol. Sci. 147 (2), 211–221. 10.1016/j.jphs.2021.06.005 34217619

[B53] Morozumi W. Aoshima K. Inagaki S. Iwata Y. Takagi Y. Nakamura S. (2022). Piezo1 activation induces fibronectin reduction and PGF2α secretion via arachidonic acid cascade. Exp. Eye Res. 215, 108917. 10.1016/j.exer.2021.108917 34973946

[B54] Ohno-Matsui K. Wu P. C. Yamashiro K. Vutipongsatorn K. Fang Y. Cheung C. M. G. (2021). IMI pathologic myopia. Invest. Ophthalmol. Vis. Sci. 62 (5), 5. 10.1167/iovs.62.5.5 33909033 PMC8083114

[B55] Overby D. R. Stamer W. D. Johnson M. (2009). The changing paradigm of outflow resistance generation: towards synergistic models of the JCT and inner wall endothelium. Exp. Eye Res. 88 (4), 656–670. 10.1016/j.exer.2008.11.033 19103197 PMC2744486

[B56] Rada J. A. Shelton S. Norton T. T. (2006). The sclera and myopia. Exp. Eye Res. 82 (2), 185–200. 10.1016/j.exer.2005.08.009 16202407

[B57] Raghunathan V. K. Morgan J. T. Dreier B. Reilly C. M. Thomasy S. M. Wood J. A. (2013). Role of substratum stiffness in modulating genes associated with extracellular matrix and mechanotransducers YAP and TAZ. Invest. Ophthalmol. Vis. Sci. 54 (1), 378–386. 10.1167/iovs.12-11007 23258147 PMC3594895

[B58] Ryskamp D. A. Frye A. M. Phuong T. T. Yarishkin O. Jo A. O. Xu Y. (2016). TRPV4 regulates calcium homeostasis, cytoskeletal remodeling, conventional outflow and intraocular pressure in the mammalian eye. Sci. Rep. 6, 30583. 10.1038/srep30583 27510430 PMC4980693

[B59] Saotome K. Murthy S. E. Kefauver J. M. Whitwam T. Patapoutian A. Ward A. B. (2018). Structure of the mechanically activated ion channel Piezo1. Nature 554 (7693), 481–486. 10.1038/nature25453 29261642 PMC6010196

[B60] Siegwart J. T. Jr. Norton T. T. (2005). Selective regulation of MMP and TIMP mRNA levels in tree shrew sclera during minus lens compensation and recovery. Invest Ophthalmol. Vis. Sci. 46 (10), 3484–3492. 10.1167/iovs.05-0194 16186323 PMC1987367

[B61] Sigal I. A. Ethier C. R. (2009). Biomechanics of the optic nerve head. Exp. Eye Res. 88 (4), 799–807. 10.1016/j.exer.2009.02.003 19217902

[B62] Sigal I. A. Flanagan J. G. Ethier C. R. (2005). Factors influencing optic nerve head biomechanics. Invest Ophthalmol. Vis. Sci. 46 (11), 4189–4199. 10.1167/iovs.05-0541 16249498

[B63] Solis A. G. Bielecki P. Steach H. R. Sharma L. Harman C. C. D. Yun S. (2019). Mechanosensation of cyclical force by PIEZO1 is essential for innate immunity. Nature 573 (7772), 69–74. 10.1038/s41586-019-1485-8 31435009 PMC6939392

[B64] Sripinun P. See L. P. Nikonov S. Chavali V. R. M. Vrathasha V. He J. (2024). Piezo1 and Piezo2 channels in retinal ganglion cells and the impact of Piezo1 stimulation on light-dependent neural activity. bioRxiv. 10.1101/2024.06.25.599602 38979351 PMC11230181

[B65] Swain S. M. Romac J. M. Vigna S. R. Liddle R. A. (2022). Piezo1-mediated stellate cell activation causes pressure-induced pancreatic fibrosis in mice. JCI Insight 7 (8), e158288. 10.1172/jci.insight.158288 35451372 PMC9089793

[B66] Syeda R. Xu J. Dubin A. E. Coste B. Mathur J. Huynh T. (2015). Chemical activation of the mechanotransduction channel Piezo1. Elife 4, e07369. 10.7554/eLife.07369 26001275 PMC4456433

[B67] Tamm E. R. (2009). The trabecular meshwork outflow pathways: structural and functional aspects. Exp. Eye Res. 88 (4), 648–655. 10.1016/j.exer.2009.02.007 19239914

[B68] Tham Y. C. Li X. Wong T. Y. Quigley H. A. Aung T. Cheng C. Y. (2014). Global prevalence of glaucoma and projections of glaucoma burden through 2040: a systematic review and meta-analysis. Ophthalmology 121 (11), 2081–2090. 10.1016/j.ophtha.2014.05.013 24974815

[B69] Troilo D. Smith E. L. Nickla D. L. Ashby R. Tkatchenko A. V. Ostrin L. A. (2019). IMI - Report on experimental models of emmetropization and myopia. Invest. Ophthalmol. Vis. Sci. 60 (3), M31–m88. 10.1167/iovs.18-25967 30817827 PMC6738517

[B70] Uchida T. Shimizu S. Yamagishi R. Tokuoka S. M. Kita Y. Honjo M. (2021). Mechanical stretch induces Ca(2+) influx and extracellular release of PGE(2) through Piezo1 activation in trabecular meshwork cells. Sci. Rep. 11 (1), 4044. 10.1038/s41598-021-83713-z 33597646 PMC7890064

[B71] van Zyl T. Yan W. McAdams A. Peng Y. R. Shekhar K. Regev A. (2020). Cell atlas of aqueous humor outflow pathways in eyes of humans and four model species provides insight into glaucoma pathogenesis. Proc. Natl. Acad. Sci. U. S. A. 117 (19), 10339–10349. 10.1073/pnas.2001250117 32341164 PMC7229661

[B72] Vranka J. A. Kelley M. J. Acott T. S. Keller K. E. (2015). Extracellular matrix in the trabecular meshwork: intraocular pressure regulation and dysregulation in glaucoma. Exp. Eye Res. 133, 112–125. 10.1016/j.exer.2014.07.014 25819459 PMC4379427

[B73] Wan Y. Wang H. Fan X. Bao J. Wu S. Liu Q. (2023). Mechanosensitive channel Piezo1 is an essential regulator in cell cycle progression of optic nerve head astrocytes. Glia 71 (5), 1233–1246. 10.1002/glia.24334 36598105

[B74] Wang S. Chennupati R. Kaur H. Iring A. Wettschureck N. Offermanns S. (2016). Endothelial cation channel PIEZO1 controls blood pressure by mediating flow-induced ATP release. J. Clin. Invest. 126 (12), 4527–4536. 10.1172/jci87343 27797339 PMC5127677

[B75] Wu H. Chen W. Zhao F. Zhou Q. Reinach P. S. Deng L. (2018). Scleral hypoxia is a target for myopia control. Proc. Natl. Acad. Sci. U. S. A. 115 (30), E7091–e7100. 10.1073/pnas.1721443115 29987045 PMC6064999

[B76] Yang C. Tibbitt M. W. Basta L. Anseth K. S. (2014). Mechanical memory and dosing influence stem cell fate. Nat. Mater. 13 (6), 645–652. 10.1038/nmat3889 24633344 PMC4031270

[B77] Yao M. Tijore A. Cheng D. Li J. V. Hariharan A. Martinac B. (2022). Force- and cell state-dependent recruitment of Piezo1 drives focal adhesion dynamics and calcium entry. Sci. Adv. 8 (45), eabo1461. 10.1126/sciadv.abo1461 36351022 PMC9645726

[B78] Yarishkin O. Phuong T. T. T. Bretz C. A. Olsen K. W. Baumann J. M. Lakk M. (2018). TREK-1 channels regulate pressure sensitivity and calcium signaling in trabecular meshwork cells. J. Gen. Physiol. 150 (12), 1660–1675. 10.1085/jgp.201812179 30446509 PMC6279358

[B79] Yarishkin O. Phuong T. T. T. Baumann J. M. De Ieso M. L. Vazquez-Chona F. Rudzitis C. N. (2021). Piezo1 channels mediate trabecular meshwork mechanotransduction and promote aqueous fluid outflow. J. Physiol. 599 (2), 571–592. 10.1113/jp281011 33226641 PMC7849624

[B80] Yuan Y. Li M. Yao Q. Qi Y. Ke B. (2026). Piezo2 mechanically regulate scleral fibroblast differentiation by activating relA/RhoA pathway. Exp. Eye Res. 267, 110955. 10.1016/j.exer.2026.110955 41780837

[B81] Zhang L. Liu Q. Yang X. Su C. Ding H. Hu J. (2025). Mechanosensitive ion channel PIEZO1 as a key regulator of intestinal fibrosis in Crohn's Disease. Inflamm. Bowel Dis. 31 (7), 1772–1788. 10.1093/ibd/izaf041 40053528

[B82] Zhao Q. Zhou H. Chi S. Wang Y. Wang J. Geng J. (2018). Structure and mechanogating mechanism of the Piezo1 channel. Nature 554 (7693), 487–492. 10.1038/nature25743 29469092

[B83] Zhao X. Kong Y. Liang B. Xu J. Lin Y. Zhou N. (2022). Mechanosensitive Piezo1 channels mediate renal fibrosis. JCI Insight 7 (7), e152330. 10.1172/jci.insight.152330 35230979 PMC9057604

[B84] Zheng M. Yao Y. Borkar N. A. Thompson M. A. Zhang E. Drake L. Y. (2024). Piezo channels modulate human lung fibroblast function. Am. J. Physiol. Lung Cell Mol. Physiol. 327 (4), L547–l556. 10.1152/ajplung.00356.2023 39189800 PMC11905809

[B85] Zhong W. Lan C. Gu Z. Tan Q. Xiang X. Zhou H. (2023). The mechanosensitive Piezo1 channel mediates mechanochemical transmission in myopic eyes. Invest. Ophthalmol. Vis. Sci. 64 (7), 1. 10.1167/iovs.64.7.1 37261388 PMC10241313

[B86] Zhu W. Hou F. Fang J. Bahrani Fard M. R. Liu Y. Ren S. (2021). The role of Piezo1 in conventional aqueous humor outflow dynamics. iScience 24 (2), 102042. 10.1016/j.isci.2021.102042 33532718 PMC7829208

[B87] Zhu Y. Garcia-Sanchez J. Dalal R. Sun Y. Kapiloff M. S. Goldberg J. L. (2023). Differential expression of PIEZO1 and PIEZO2 mechanosensitive channels in ocular tissues implicates diverse functional roles. Exp. Eye Res. 236, 109675. 10.1016/j.exer.2023.109675 37820892 PMC10843266

[B88] Zhu L. Ren S. He S. Liu M. Hou F. (2025). Piezo in the eye: expression, distribution and roles in ocular diseases. Front. Physiol. 16, 1651258. 10.3389/fphys.2025.1651258 40873759 PMC12378635

